# The Impact of Different Oxygen Delivery Methods on Corneal Epithelial Repair after Injury

**DOI:** 10.1155/2022/3260087

**Published:** 2022-10-03

**Authors:** Shanshan Li, Qingfen Tian, Gang Ding, Yuqin Sun, Zhongkai Hao, Xu Wang, Chenming Zhang, Yuan Tao

**Affiliations:** ^1^Jinan Second People's Hospital (Jinan Eye Hospital), Jinan 250001, China; ^2^Department of Ophthalmology, Affiliated Hospital of Weifang Medical University, Weifang 261000, China

## Abstract

The hyperbaric oxygen therapy is often used in the management of acid and base burns of the eyes. However, oxygen is rarely supplied locally through goggles or face mask in ophthalmology. Therefore, in this study, we aim to investigate how oxygen delivery affects eye recovery after injury. We used a rabbit model with corneal epithelial injury to examine the effects of local oxygen supply via goggles or face mask on the recovery of cornea. A total of 75 healthy New Zealand white rabbits were randomly divided into three groups, A, B, and C, with 25 rabbits in each group. Then, on each rabbit eye (150 eyes in total), a circle of corneal epithelium with 5 mm in diameter was scraped off from the center of the cornea with a corneal epithelial scraper. Group A was given oxygen goggles every day (the oxygen flow rate was 3 L/min, once a day, 2 hours each time); group B was given nasal inhalation of oxygen every day (the oxygen flow rate was 3 L/min, once a day, 2 hours each time); and group C did not receive any treatment and was healed naturally. We found that the group A, which received oxygen supply via goggles, showed the best eye recovery. Transmission electron microscopy showed that the cornea with local oxygen supply via goggles or face mask exhibited intact capillary structure and obvious desmosome/hemidesmosome connections between cells. Moreover, the protein and RNA levels of hypoxia-related genes were lower in group A and B, suggesting that the hypoxia factor is a sensitive and early regulator in the low oxygen environment.

## 1. Introduction

In recent years, the incidence of ocular surface diseases has been increasing, and the corneal epithelium is the first barrier to be penetrated by the pathogens. Rapid repair of corneal epithelium after injury is the most important condition for maintaining the physiological functions of cornea [1]. Oxygen plays an important role in the process of corneal injury and repair. For example, hypoxia can slow down the corneal repair process [2] and can also lead to the apoptosis of normal corneal epithelial cells [3]. During the repair of corneal epithelial injury, the changes in ultrastructures such as microvilli and matrix proteins in the stroma are most obvious [4]. Oxygen therapy has been used in clinics for many years [5]. The most commonly used oxygen delivery methods in clinics are traditional noninvasive positive pressure ventilation (connecting patients with noninvasive ventilators through noninvasive methods such as nasal or face mask) and hyperbaric oxygen therapy (HBOT) (patients inhale oxygen in a hyperbaric environment). These methods are generally used for the treatment of respiratory diseases, and HBOT has also been used for the treatment of thermal burns [6], but there are only a few reports regarding its use in ophthalmology. In clinical ophthalmology, HBOT is more commonly used in the treatment of corneal chemical burns and scleritis [7–9]. In this study, we delivered oxygen to injured corneal epithelium using two different methods: goggles and nasal inhalation, and assessed the effects of different oxygen delivery methods on the recovery of corneal epithelium.

## 2. Materials and Methods

### 2.1. Materials

This study included 75 healthy rabbits, with a total of 150 eyes. All rabbits were New Zealand white rabbits, weighing about 2–2.5 kg (the weight difference was not statistically significant).

#### 2.1.1. Low Flow Oxygen, Goggles, and Face Mask

The reagents used in this study included: 2.5% glutaraldehyde phosphate buffer (Shanghai Luzhen Co., Ltd.); 1M PBS Buffer, pH 7.2, 1% osmic acid (Zhengzhou Ruichang Chemical Products Co., Ltd.); anhydrous acetone (Zhengzhou Ruichang Chemical Products Co., Ltd.); uranium acetate and lead citrate (Zhengzhou Ruichang Chemical Products Co., Ltd.); and Epon812 embedding media (US app company).

### 2.2. Methods

The rabbit eyes of A, B, and C groups were marked with 5 mm trephine to mark the cornea, and the middle cornea with 5 mm in diameter was scraped using corneal epithelial scraper, 2 hours at a time. The eyes in group C were exposed to the air and healed naturally. The three groups were given ofloxacin eye drop four times a day to prevent infection. Corneal fluorescein staining was performed at 12 h, 24 h, 36 h, 48 h, 60 h, and 72 h after injury. At 24 h after injury, 5 rabbits with 10 eyes were randomly selected from each group and sacrificed by air embolization. The corneas of the rabbits were collected, and the corneas were divided into two parts along the diameter: (1). Half of the corneal tissue specimen was placed in 2.5% glutaraldehyde phosphate buffer within 30 seconds and stored at 4°C; the other half was stored in −80°C freezer for protein and RNA measurements of hypoxia factors. (2). When selecting specimens: half of the corneal tissue specimen was put in the wax box: about 1 × 1 × 3 mm corneal specimen from the center of the cornea was taken and immersed in 2.5% glutaraldehyde phosphate buffer within 30 seconds. (3). The specimen was rinsed, fixed, and rinsed again; then, the specimen was dehydrated, infiltrated, embed, and sectioned into ultrathin slices. 5. The sections were stained with citric acid and uranyl acetate, respectively, and washed three times; then, the sections were dried and examined under transmission electron microscope. At 48 hours after injury, 5 rabbits with 10 eyes were randomly selected from each group and sacrificed by air embolization; and their corneas were collected for protein and RNA measurements of hypoxia factors. At 72 hours after injury, 5 rabbits from each group were sacrificed by air embolization, and their corneas were collected for pathological examination. The remaining 15 rabbits in each group, a total of 30 eyes, received continuous corneal fluorescein staining at 12 h, 24 h, 36 h, 48 h, 60 h, and 72 h after injury to examine the recovery of corneal epithelium.

### 2.3. Statistical Analysis

(1) Kruskal–Wallis H rank sum test was used to compare the number of corneal epithelial cell layers between different groups. (2) Chi-square test was used to compare the protein and RNA levels of hypoxia factor in corneal epithelium at 12 h and 24 h after injury. All data were expressed as mean ± standard deviation. For overall comparison, *P* < 0.05 was considered statistically significant; because of the multiple comparisons among multiple sample rates, in order to avoid error I, it is suggested to change the level to *P*=0.05/3=0.017. For pairwise comparison, *P* < 0.017 was considered statistically significant.

## 3. Results

### 3.1. Corneal Epithelium Staining

**Figure 1:**
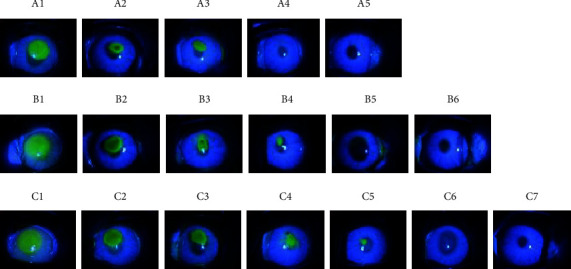
Corneal staining analysis in each group at 0 h, 12 h, 36 h, 48 h, 60 h, and 72 h after operation. Group A: 0 h (A1), 12 h (A2), 24 h (A3), 36 h (A4), 48 h (A5); Group B: 0 h (B1), 12 h (B2), 24 h (B3), 36 h (B4), 48 h (B5), 60 h (B6); Group C: 0 h (C1), 12 h (C2), 24 h (C3), 36 h (C4), 48 h (C5), 60 h (C6), 72 h (C7). The corneal fluorescence staining at 12 h, 24 h, 36 h, 48 h, and 72 h after operation: the staining area of group A was significantly smaller than group B at each time point, and the staining area of group B was smaller than group C.

The staining area in three groups at each time point followed the order of group A < group B < group C, suggesting that the corneal epithelium healing was the fastest in group A, followed by group B, and slowest in group C (Figure 1).

**Figure 2:**
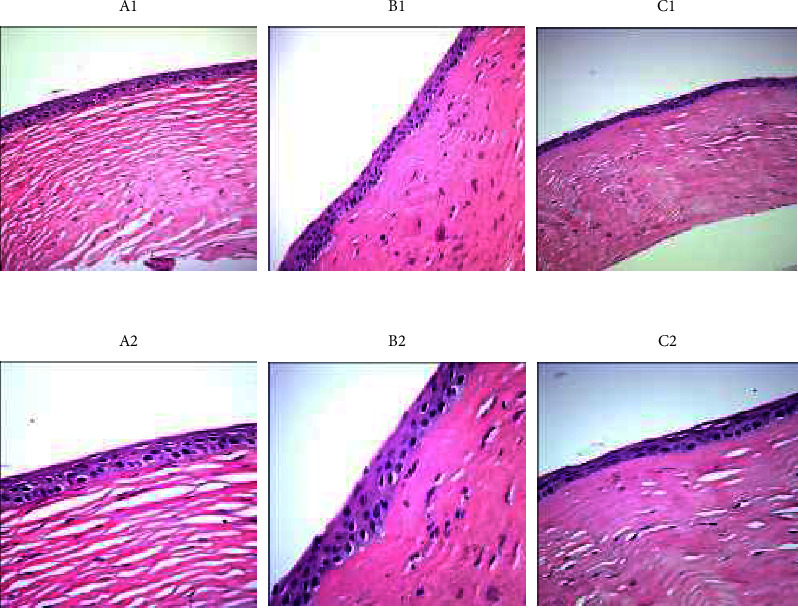
Electron microscope images in each group at 72 h after operation. Electron microscopic images of group A (A1), B (B1), and C (C1) under 200x magnification; electron microscopic images of group A (A2), B (B2), and C (C2) under 400x magnification. Group A: there are 5–6 layers of corneal epithelium; no obvious neovascularization is seen at corneal limbus; the matrix collagen structure is densely and regularly arranged. Group B: there are 4–5 layers of corneal epithelium; new blood vessels can be seen at the corneal limbus; the matrix collagen structure is densely and regularly arranged. Group C: there are 2–3 layers of corneal epithelium; a large number of new blood vessels can be seen at the corneal limbus; the number of fibroblasts in the stromal layer is reduced, and a large number of inflammatory cells are observed; matrix collagen structure is slightly loose but relatively regular.

The protein and RNA levels of hypoxia factor at 24 hours and 48 hours after injury followed the order of group A < group B < group C (*P* < 0.05, Figures 1 and 2), demonstrating that at both 24 and 48 hours after injury, the protein and RNA levels of hypoxia factor were the lowest in the oxygen goggle group, followed by the oxygen inhalation group, and the highest in the control group.

### 3.2. The Pathology Examination at 72 Hours after Injury and the Number of Corneal Epithelial Cell Layers in Three Groups

Group A: the cornea was smooth and thicker in group A compared to group C. There were 4 layers of epithelium. No obvious neovascularization was found in the corneal limbus. The matrix collagen structure was densely and regularly arranged. Group B: the cornea in group B was smooth and slightly thicker than the control group, with 3–4 layers of epithelium. New blood vessels can be seen in the corneal limbus. The matrix collagen structure was densely and regularly arranged. Group C: the cornea of group C was smooth and slightly thinner than the experimental groups, with 2–3 layers of epithelium. A large number of new blood vessels can be seen in the corneal limbus. The number of fibroblasts in the stromal layer was reduced, and many inflammatory cells were observed. The matrix collagen structure was slightly loose, but still regular (Figure 2). The differences between A, B, and C groups were statistically significant, and the number of corneal epithelial cell layers after 72 hours of intervention was the highest in group A, followed by group B, and the lowest in group C.

### 3.3. Transmission Electron Microscopy

**Figure 3:**
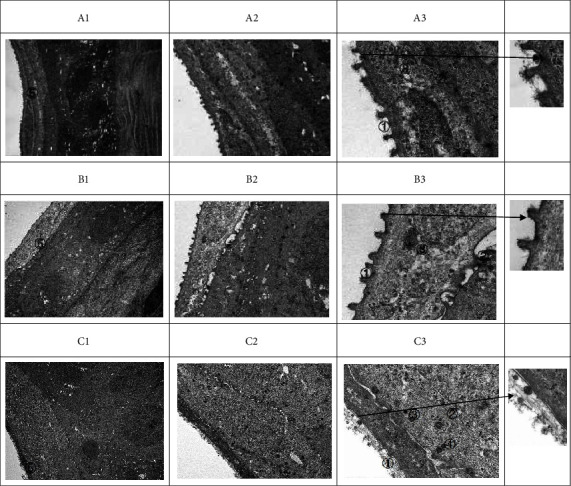
TEM images in each group at 24 h after operation. TEM images of group A: 5,000x (A1), 15,000x (A2), and 50,000x (A3); TEM images of group B: 5,000x (B1), 15,000x B1 (B2), and 50,000x (B3); TEM images of group C: 5,000x (C1), 15,000x (C2), and 50,000x (C3). (1) The microvilli in A and B groups are closely and regularly arranged. The microvilli in group C are absent, and the surface epithelial cells are necrotic (①). (2) There are a lot of glycocalyx on the cell membrane of microvilli in A and B groups, but no glycocalyx is seen in in group C (①, the enlarged picture is indicated by the arrow). (3) Desmosome junction and hemidesmosome junction can be seen in A and B groups; in group (C), the distribution of desmosome junctions is disordered, the number of desmosomes is reduced, the tension filaments are significantly reduced, and necrotic cells can be seen on one side (②). (4) In group B and C, the mitochondria are swollen, endoplasmic reticulum is expanded, and mitochondrial cristae are damaged (③); myeloid corpuscles are visible. (5) The number of corneal epithelium layers in group C is more than that in group A and B under transmission electron microscope (④). (6) The corneal epithelium layers in group C were more than that in group A and B, but the boundary of each stratification is not obvious (⑤).

Transmission electron microscopy analysis:

- The microvilli of A and B groups were tightly and regularly arranged. In group C, the microvilli were damaged and the surface epithelial cells were necrotic (① in Figure 3).
- Glycocalyx could be seen on microvillus cell membranes in A and B groups, and group A had more glycocalyx; on the contrary, no glycocalyx was observed in group C (① in Figure 3, arrow indicates the enlarged microvilli).
- Obvious desmosome connection and hemidesmosome connection can be seen in A and B groups. In group C, the distribution of desmosome junctions was disordered, the number was reduced, the tension filaments were significantly reduced, and necrotic cells could be seen on one side (② in Figure 3).
- In group C and B, the mitochondria of epithelial cells were swollen, the endoplasmic reticulum was expanded, the mitochondrial cristae were damaged (③ in Figure 3), and the myeloid bodies were visible (④ in Figure 3).
- Under transmission electron microscope, the corneal epithelium layers in group C were more than that in group A and B, but the boundary of each stratification is not obvious (⑤ in Figure 3). In group C, the corneal epithelial cells were obviously swollen; their endoplasmic reticulum was expanded, the mitochondrial cristae were damaged, and myeloid corpuscles were visible (④ in Figure 3).

## 4. Discussion

The corneal epithelium is located in the outermost layer of the cornea. Because of its ability to repair quickly after injury, the corneal epithelium plays a key role in preventing the invasion of pathogens and resisting damage [10]. Corneal epithelial cells can regulate corneal injury repair, corneal cell apoptosis, myofibroblast transdifferentiation, and corneal neovascularization [11–13]. Early epithelial healing can prevent long-term complications with persistent corneal epithelial defects, such as bacterial superinfection, endophthalmitis, and symblepharon formation. Therefore, how to improve the rapid repair of corneal epithelium is of great significance in the treatment of eye diseases.

In normal organisms, oxygen concentration plays an important role in regulating the metabolism, proliferation, and differentiation of tissue cells [14]. Cornea is a nonvascular tissue, and thus it mainly depends on atmospheric oxygen dissolved in aqueous humor and oxygen contained in tears to generate ATP through oxidative phosphorylation [15]. Therefore, oxygen levels are important for the regulation of cellular metabolism and fluctuations in corneal epithelium, as well as the stability and expression of proteins [16].

Oxygen promotes corneal wound healing and functional recovery through a variety of mechanisms, including upregulation of growth factors, modulation of inflammatory cytokines, alleviation of edema, and improvement of leukocyte function. In addition, oxygen exhibits antibacterial effects and supports new tissue growth [17, 18]. During the process of corneal epithelial damage repair, the changes of ultrastructures such as microvilli and matrix proteins in the stroma are the most obvious [4]. In our study, the pathological examination showed that (Figure 2): the structure of matrix collagen in group A and B was densely and regularly arranged, while in group C, the matrix collagen structure was loosely arranged. Although oxygenation of hypoxic tissue is a key mechanism in accelerating wound healing, oxygen also affects wound repair in numerous ways after the termination of treatment [17, 18]. This also explains why in our experiment, the epithelial stratification in group C was significantly more than that of group A and B under transmission electron microscope at 24 hours after intervention (Figure 3); but at 72 hours after intervention, the epithelial stratification in group C was significantly less than group A and B.

The complete repair of corneal epithelium requires three conditions: epithelial cell regeneration, migration, and adhesion. Among them, the adhesion of corneal epithelial cells refers to the tight junctions between epithelial cells and the desmosome and hemidesmosome structures between epithelial cells and basement membrane [19]. The maintenance of the normal corneal epithelial structure requires the adsorption of various components in tear film by the microvilli, especially the mucus. In our study, transmission electron microscopy showed that (Figure 3), in A and B groups, which were given oxygen in different ways, the microvilli were closely arranged and intact; moreover, a large number of glycocalyx were found between the microvilli, and many desmosome connections and hemidesmosome connections could be seen. On the contrary, in group C, microvilli were absent and surface epithelial cells were necrotic; the numbers of desmosome connections and tension filaments were significantly reduced. However, it is still unclear how oxygen stimulates biological processes such as angiogenesis, collagen synthesis, and vascular endothelial growth factor release under hypoxia and hyperoxia [17]. It has been proposed that the effects of oxygen are related to its promotion of acetylcholine content in the corneal epithelium [20]. Acetylcholine can bind to the *α*7nAChR on macrophage surface and further inhibit the production and release of inflammatory factors through intracellular JAK2-STAT3, NF-*κ*B, and other signal transduction pathways, which reduces local or systemic inflammatory responses [21–23]. It is known that oxygen can reduce collagen synthesis by tissue fibroblasts, thereby reducing the leukocyte's ability of killing bacteria; these effects then lead to increased corneal epithelialization and decreased limbal stem cell destruction [24]. Thus, oxygen plays a role in reducing inflammation by regulating related cytokines and tumor necrosis factor [4]. In this study, the structure of matrix collagen in the oxygen intervention group (groups A and B) was densely arranged, no obvious inflammatory cells were found, and the corneal epithelium appeared normal, all of which could be due to the effects of oxygen mentioned above (Figure 2 and Table 1).

The different gene expression profiles under normoxia and hypoxia conditions can lead to differences in cell proliferation ability [25]. In this study, we found that: the number of corneal epithelium was 4 in group A and 3–4 in group B, and the corneas of both groups were smooth and slightly thicker than that of the control group; in group C, due to the lack of oxygen supply probably, the corneal epithelium only had 2–3 layers (Figure 2). It is worth to be noted that, at 72 hours after injury, the number of corneal epithelial cell layers in group A was higher than that in group B and significantly higher than that in group C (Table 1), suggesting that increasing the local oxygen supply to the eye can significantly enhance the proliferation ability of cells in all layers. However, the transmission electron microscopy at 24 hours after injury showed that the corneal epithelium in group C had significantly more cell layers than group A and B under 5000x and 15000x transmission electron microscopy, but the boundaries between each layer were blurry, the cells were swollen with expanded endoplasmic reticulum, the mitochondrial crest was damaged, and the myeloid bodies were visible (Figure 3). This phenomenon might be related to hypoxia-inducible factor-1a (HIF-1a), which is higher in the corneal limbus [26]. HIF-1 is the main oxygen regulator composed of HIF-1*α* and HIF-1*β*. It participates in the regulation of cell survival, apoptosis, energy metabolism, oxygen homeostasis, and other cellular functions by affecting the expression of more than 200 genes. HIF-1*α* is an early oxygen regulator that is more sensitive to hypoxia, and it is stimulated by hypoxia or other cytokines [27–29]. However, HIF-1*α* is easily degraded via ubiquitin pathway in the cornea under normoxic state, and thereby its expression level is low. Under hypoxic state, HIF-1*α* activates the expression of various hypoxia response factors in cells by binding to the hypoxia response element (HRE) of related genes. Therefore, HIF-1*α* can promote the differentiation and swelling of corneal epithelial cells and increase cell stratification with blurred boundaries [30, 31]. This is also consistent with our measurements on protein and RNA levels of hypoxia factor (Tables 1 and 2). In addition, HIF-1*α* mostly exists in corneal limbal stem cells. If the hypoxic environment persists, the hypoxic microenvironment may produce cascade effects by stimulating other tissue factors or stimulating paracrine secretion from other cells and tissues. Also, the damage effect of long-term hypoxic microenvironment on stem cells and tissue cells is also an unavoidable problem, which can explain that the stratification in group C was significantly lower than that in group A and B at 72 hours after injury (Figure 2).

In this study, we found that increasing the oxygen concentration in the surrounding environment of the eye can significantly enhance the proliferation ability of corneal epithelial cells and shorten the recovery time after corneal epithelial injury, providing a theoretical basis for corneal epithelial injury in clinics. Based on our findings, early oxygen therapy is of great significance to promote corneal epithelial recovery. However, due to the short observation time, whether different oxygen concentrations and different oxygen supply time will affect corneal epithelium repair, and whether the corneal epithelium will be damaged again after repairing need further investigation and exploration. The current study is an animal study, and that the conclusions may not apply to other species (particularly humans).

## Figures and Tables

**Table 1:** The protein levels of hypoxia factor.

	24 hour	48 hour	*t*	*P*
Group A (*n* = 10)	0.73 ± 0.26	0.12 ± 0.09	6.7846	0.0001
Group B (*n* = 10)	0.97 ± 0.08	0.52 ± 0.14	7.7607	0.0000
Group C (*n* = 10)	0.79 ± 0.04	0.37 ± 0.14	13.4526	0.0000
*F*	6.35	27.63		
*P*	0.0055	0.0000		

At 24 and 48 hours after corneal epithelial intervention, the protein levels of hypoxia factor in three groups were: group A < group B < group C, the difference was statistically significant.

**Table 2:** The RNA levels of hypoxia factor.

	24 hour	48 hour	*t*	*P*
Group A (*n* = 10)	0.46 ± 0.31	0.16 ± 0.06	2.9061	0.0174
Group B (*n* = 10)	0.37 ± 0.23	0.06 ± 0.05	3.9773	0.0032
Group C (*n* = 10)	0.79 ± 0.19	0.37 ± 0.05	6.3254	0.0001
*F*	8.01	101.08		
*P*	0.0019	0.0000		

At 24 and 48 hours after corneal epithelial intervention, the RNA levels of hypoxia factor in three groups were: group A < group B < group C, the difference was statistically significant.

## Data Availability

All data generated or analyzed during this study are available from the corresponding author Chenming Zhang upon reasonable request.
